# Supplementary material for: Comparative Efficacy and Safety of P2Y12 Inhibitor Monotherapy and Dual Antiplatelet Therapy in Patients with and without Diabetes Mellitus Undergoing Percutaneous Coronary Intervention
Source: Int J Mol Sci. 2022 Apr 20;23(9):4549. doi: 10.3390/ijms23094549 (PMC9099862; doi:10.3390/ijms23094549)
Supplement: Supplementary file 1 [file ijms-23-04549-s001.zip › ijms-1685233-supplementary.pdf]

Table S1. EMBASE search strategy.

- The Embase database: Contains biomedical literature from 1974 to present.
- The MEDLINE & PubMed database: Covers journals from 1966 to present.
- Embase Classic: The Embase back file covering almost 2 million biomedical and pharmacological citations drawn from over 3,000 international titles from between 1947 and 1973

Table S2. PubMed search strategy.

Search	Query	Results
#1	'percutaneous coronary intervention':ab,ti OR 'percutaneous coronary revascularization':ab,ti OR pci:ab,ti OR (('percutaneous coronary' NEAR/3 intervention):ab,ti) OR (('percutaneous coronary' NEAR/3 revascularization):ab,ti) OR 'drug eluting stents':ab,ti OR 'drug-eluting stents':ab,ti OR des:ab,ti	150,565
#2	'coronary artery disease':ab,ti OR 'myocardial infarc*':ab,ti OR 'diabetes mellitus':ab,ti OR acs:ab,ti OR cad:ab,ti OR 'cardiovascular event':ab,ti	501,041
#3	#1 AND #2	60,838
#4	'dual antiplatelet therapy':ab,ti OR 'dual anti-platelet therapies':ab,ti OR 'dapt'/exp OR dapt	12,686
#5	'monotherapy with p2y12 inhibitor':ab,ti OR 'p2y12 inhibitor monotherapy':ab,ti OR 'shot term p2y12 inhibitor':ab,ti OR 'shot-term p2y12 inhibitor':ab,ti OR clopidogrel:ab,ti OR prasugrel:ab,ti OR ticagrelor:ab,ti OR ticlopidine:ab,ti OR 'clopidogrel monotherapy':ab,ti OR 'ticagrelor monotherapy':ab,ti	30,805
#6	#4 AND #5	4,837
#7	#3 AND #6	1,762
#8	'clinical trial'/de OR 'randomized controlled trial'/de OR 'randomization'/de OR 'single blind procedure'/de OR 'double blind procedure'/de OR 'crossover procedure'/de OR ('randomized controlled' NEXT/1 trial*) OR rct OR 'randomly allocated' OR 'allocated randomly' OR 'random allocation' OR (allocated NEAR/2 random) OR (single NEXT/1 blind*) OR (double NEXT/1 blind*) OR ((treble OR triple) NEAR/1 blind*)	1,883,445
#9	'meta analysis':ab,ti OR 'systematic review':ab,ti OR sr:ab,ti	465,107
#10	'net work':ab,ti OR network:ab,ti	548,040
#11	#9 OR #10	997,217
#12	#7 AND #8 NOT #11	447

#	Searches	Results

**#1**	"percutaneous coronary intervention"[Mesh] OR "coronary intervention*, percutaneous":[tiab] OR "intervention*, percutane-ous coronary":[tiab] OR "pci":[tiab] OR "percutaneous coronary revascularization":[tiab] OR "coronary revascularization*, percu-taneous":[tiab] OR "percutaneous coronary revascularizations":[tiab] OR "revascularization*, percutaneous coronary":[tiab] OR "drug eluting stent*":[tiab] OR "des":[tiab]	**98640**
**#2**	"Dual Anti-Platelet Therapy"[Mesh] OR "dual antiplatelet therap-y":[tiab] OR "dapt":[tiab] OR "aspirin":[tiab] OR "clopidogrel":[tiab] OR "prasugrel":[tiab] OR "ticagrelor":[tiab] OR "p2y12 in-hibitor":[tiab] OR "anti-platelet therapies, dual":[tiab] OR "anti-platelet therapy, dual":[tiab] OR "dual anti-platelet therapy":[ti-ab] OR "dual anti-platelet therapies":[tiab]	**63387**
**#3**	"Randomized Controlled Trial"[Publication Type] OR "controll-ed clinical trial":[tiab] OR "clinical trials, randomized":[tiab] OR "trials, randomized clinical":[tiab] OR "randomized clinical studies":[tiab]	**564928**
**#4**	"Systematic Review"[Publication Type] OR "Meta-Analysis"[Publication Type] OR "Review"[Publication Type] OR "meta-analysis":[ti] OR "systematic review":[ti] OR "literature review":[ti] OR "expert consensus":[ti] OR "case report":[ti]	**3375129**
**#5**	**#1 AND #2 AND #3 NOT #4**	**1054**

Table S3. Cochrane Library search strategy.

#	**Searches**	**Results**
**#1**	MeSH descriptor percutaneous coronary intervention explode all trees OR (coronary intervention*, percutaneous):ti,ab,kw OR (intervention*, percutaneous coronary):ti,ab,kw OR (pci):ti,ab,kw OR (percutaneous coronary revascularization*):ti,ab,kw OR (coronary revascularization*, percutaneous):ti,ab,kw OR (revascularization*, percutaneous coronary):ti,ab,kw OR (drug eluting stent*):ti,ab,kw OR (drug-eluting stent):ti,ab,kw OR (des):ti,ab,kw	**16744**
**#2**	(dual antiplatelet therapy):ti,ab,kw OR (dapt):ti,ab,kw OR (aspirin):ti,ab,kw OR (clopidogrel):ti,ab,kw OR (prasugrel):ti,ab,kw OR (ticagrelor):ti,ab,kw OR (p2y12 inhibitor):ti,ab,kw OR (anti-platelet therapies, dual):ti,ab,kw OR (anti-platelet therapy, dual):ti,ab,kw OR (dual anti-platelet therapy):ti,ab,kw OR (dual anti-platelet therapies):ti,ab,kw	**19073**

#3	'MeSH descriptor randomized controlled trial explode all trees OR (controlled clinical trial):ti,ab OR (clinical trials, randomized):ti,ab OR (trials, randomized clinical):ti,ab OR (randomized clinical studies):ti,ab	**220299**
**#4**	**#1 AND #2 AND #3**	**678**

Table S4. Bias risk assessment of the studies.

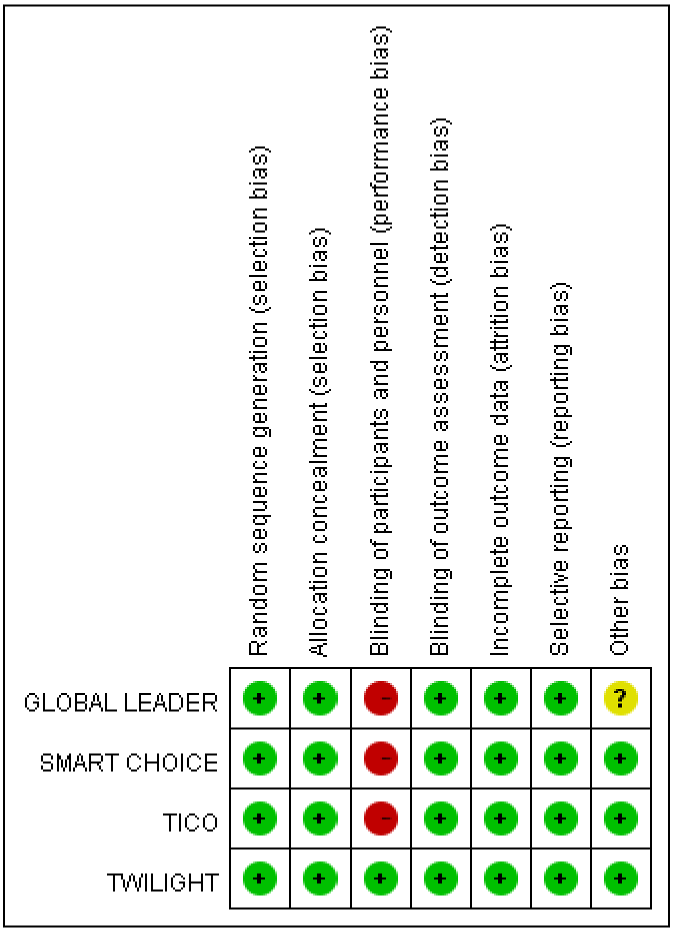

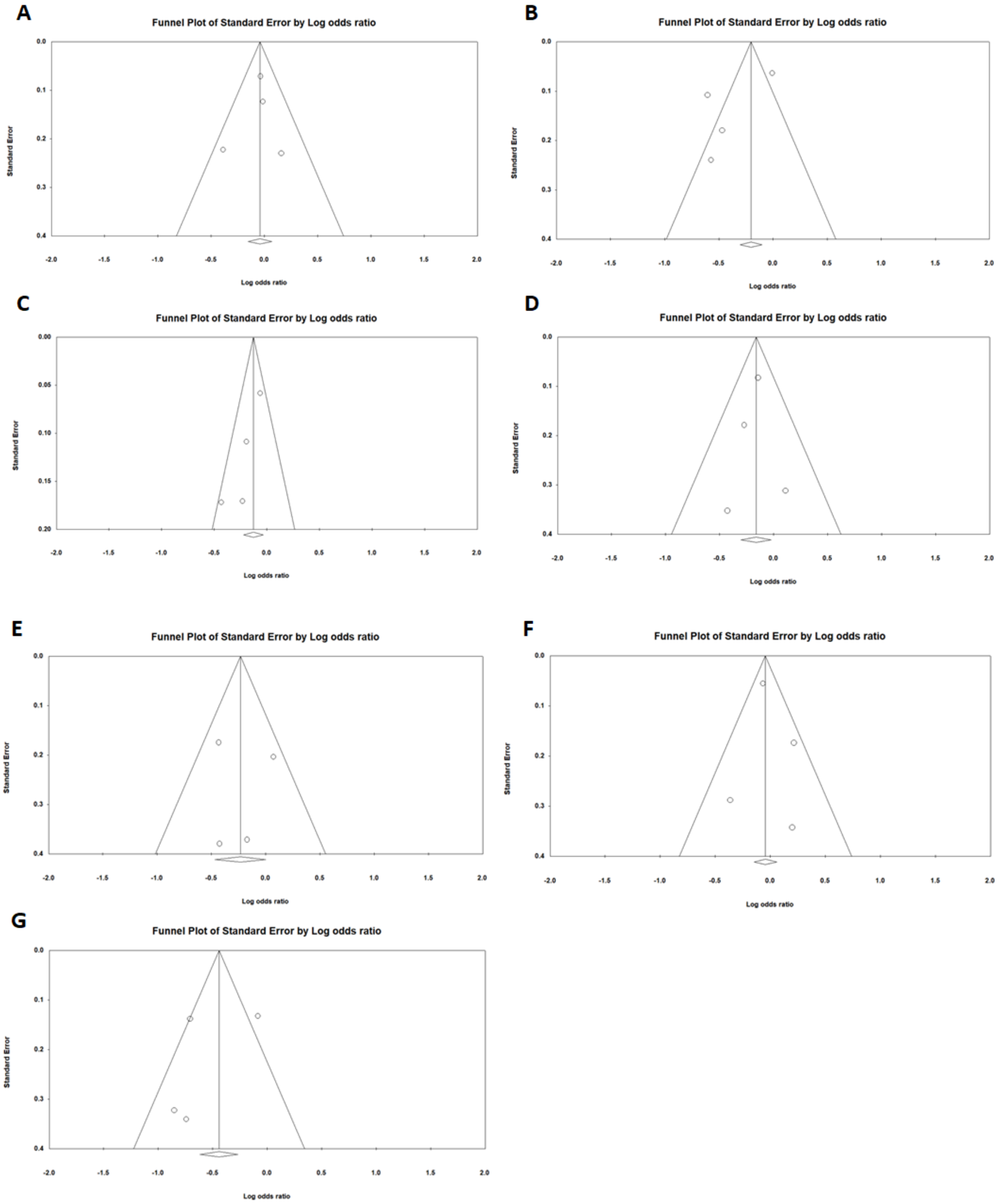

Figure S1. The funnel plots of each outcome. (**A**) MACCE, (**B**) major or bleeding, (**C**) NACE, (**D**) MACCE in DM patients, (**E**) major or minor bleeding in DM patients, (**F**) MACCE in non-DM patients, (**G**) major or minor bleeding in non-DM patients.

Table S5. The Begg's and Egger's test of each outcome.

**Endpoints**	***P*** **(Begg’s test)**	***P*** **(Egger’s test)**
MACCE	0.734	0.784

Major or minor bleeding	0.734	0.253
NACE	0.308	0.069
MACCE – DM	0.734	0.814
Major or minor bleeding – DM	1.000	0.973
MACCE – non-DM	1.000	0.719
Major or minor bleeding – non-DM	1.000	0.518
